# Coproduction for feasibility and pilot randomised controlled trials: learning outcomes for community partners, service users and the research team

**DOI:** 10.1186/s40900-018-0116-0

**Published:** 2018-10-08

**Authors:** Tracey McConnell, Paul Best, Gavin Davidson, Tom McEneaney, Cherry Cantrell, Mark Tully

**Affiliations:** 1 School of Social Science, Education and Social Work, Queen’s University Belfast, Belfast, Northern Ireland; 2 AWARE NI, Belfast, Northern Ireland; 3 School of Medicine, Dentistry and Biomedical Sciences, Queen’s University Belfast, Belfast, Northern Ireland

**Keywords:** Coproduction, Community, Partners, Challenges, Learning

## Abstract

**Plain English summary:**

Co-producing research with members of the public is increasingly recognised as a valuable process. Yet, despite these good intentions, the literature on coproduction has struggled to keep pace with the coproduction ‘movement’. There is a lack of clarity regarding acceptable levels of involvement and attempts at standardising approaches appear generic and lack detail. Moreover, relatively little research has captured the views of all the parties involved (academics, service providers and service users).

We conducted interviews with all those involved in developing a new online service for depression in Northern Ireland. Our main questions related to how these three very different groups of people worked together over a two-year period to design, develop and deliver the service (e.g. what were the benefits? What would they do differently?)

We found that early involvement was a key factor as this promoted equal ownership. There was also a need to be flexible and recognise other workload pressures. Interestingly, service providers and service users were keen to become more involved in data analysis – this is one of the most under-researched and reported areas within the coproduction literature. Finally, we considered how user involvement worked within complex research designs and how this could be improved. Based on this learning, the paper concludes with a simple 3-step framework that others may wish to follow in order to improve coproduction outcomes within interventions.

**Abstract:**

**Background**

Co-production, involving members of the public in research, is increasingly encouraged by research funders. However, reports detailing involvement of the public in the entire research process from design, delivery, analysis and dissemination of findings are lacking. Furthermore, little is known about the lessons learnt from the perspective of the public *and* researchers; or more specifically lessons learnt when coproducing specific types of research projects, such as feasibility/pilot studies incorporating a randomised controlled trial (RCT) design. This paper aims to provide a more rounded picture of co-production based on the learning outcomes of researchers, their community partners and service users involved in a feasibility/pilot RCT study developing and evaluating an E-health Service for adults with depression.

**Methods**

Qualitative research incorporating 11 semi-structured interviews with academic team members (*n* = 4), community partners (*n* = 3) and service users with depression (*n* = 4) Data were analysed using thematic analysis.

**Results**

Key factors for successful coproduction include - (1) early involvement at the pre-development stage, including contributing to the scientific grant application; (2) early identification of team strengths and expertise from the outset; (3) regular team meetings and contact (formal or informal) among coproduction partners; (4) a flexible and pragmatic approach to research design (particularly within RCTs); (5) shared decision making and responsibility and (6) recognition of ‘other’ pressures and providing support to each other. Findings also suggested further scope for involving community partners in data analysis and dissemination through co-authored papers. Those seeking to coproduce interventions or utilise RCT designs should consider tensions between data quality and intervention implementation and ethical issues regarding control groups.

**Conclusion**

This paper confirms previous research confirming the benefits of coproduction. However, it also highlights a number of barriers, particularly when using complex research design, such as RCTs. Learning points are summarised in an implementation model for coproducing research. This model may provide a useful guide for considering activities associated with meaningful coproduction. We urge others to test this proposed model more widely in different areas of coproduced research.

**Electronic supplementary material:**

The online version of this article (10.1186/s40900-018-0116-0) contains supplementary material, which is available to authorized users.

## Background

Coproduction as a concept was proposed by Ostrom and colleagues towards the end of the 1970s [1]. They defined coproduction as ‘a process through which inputs used to produce a good or service are contributed by individuals who are not “in” the same organisation’ (p. 1073) [1].

More recently, the term ‘coproduction’ has increasingly been used in relation to the involvement of service users, the public, healthcare practitioners, and community partners (i.e. representatives of community organisations) in research. [2–4] Research in this area has grown rapidly, and hence the terms ‘coproduction’ and ‘patient and public involvement’ (PPI), including patients, service users, survivors, carers and family members)) have become more widespread in the academic literature [5–7]. The exponential growth of PPI involvement in research can be attributed to the now established recognition that involving patients and the public produces higher quality research through providing unique insights into patient and public needs [7]. This includes the generation of new ideas and solutions to complex problems by offering an ‘insider’ perspective. As such PPI avoids wasting research funding, by ensuring research answers questions and measure outcomes of relevance and importance to service users [8].

A number of models have been developed to help researchers and the public assess the quality of PPI in research. Morrow et al. [9] developed a Quality Involvement Framework and a Quality Involvement Questionnaire with the aim of providing a more nuanced understanding of the processes and outcomes that demonstrate quality PPI. Staniszewska et al. [10] also developed the GRIPP (Guidance for Reporting Involvement of Patients and Public) checklist to further support and encourage researchers to provide a detailed report of PPI in their research. This checklist was developed with the additional aim of strengthening the evidence base for PPI, thereby enabling evaluations of what works in relation to PPI, for whom, in what contexts and why.

The National Institute for Health Research have also produced standards for PPI in research which provides a framework for researchers to consider how best to involve the public, and for the public to understand what their involvement may entail [11]. These standards emphasize the importance of using a ‘coproduction’ model whereby the public are involved as ‘co-researchers’, and valued as equal members within the research team [12].

However, these frameworks, checklists and standards do not include a comprehensive picture of the experiences of *all* potential PPI partners, such as community partners, service users and researchers. While some studies have shown how user involvement has influenced the thinking and attitudes of researchers [13], exploring the experience of *all involved parties* is currently missing. This is vital for facilitating a more nuanced understanding of learning across different partners. Furthermore, while coproduction of research is characterised by involvement at all stages of the research process [12] the fidelity to this model is challenging. Are researchers willing to compromise with regards to methodological rigour in order to facilitate ‘real world’ implementation difficulties? Some have argued that the power held by researchers often results in tokenistic behaviour, whereby user involvement is a rubber stamping process [14, 15]. The extent to which pragmatic decisions regarding implementation take precedence over research design and data quality are rarely explored within this literature.

Boyle et al. [16] have carried out extensive research on how to apply coproduction to public services based on learning from over 100 practitioners’ experiences, insights, challenges and successes. This large project has helped produce a framework including six guiding principles of coproduction: Recognising people as assets (from passive recipients to equal partners); Building on people’s existing capabilities (people’s abilities recognised and utilised); Promoting mutuality and reciprocity (offering incentives and enabling mutual responsibilities); Developing peer networks (between public and professionals to transfer knowledge and support change); Breaking down barriers between professionals and recipients; Facilitating rather than simply delivering service development.

Although initially formulated to conceptualise relationships between those in power of public services and public service users, coproduction theory and principles have more recently been used in the context of increasing collaborations between policy makers and practitioners in the application of health research [17–19].

There is also an emerging evidence base on how to optimise coproduction with community partners in terms of what works [4, 20], and with service users in terms of developing strategies to aid analysis of findings in partnership with non-academics [21, 22]. There has also been an emerging body of knowledge in relation to the impact of coproduction on service users and on research outcomes [23–26]. The most commonly reported impact on research outcomes include shaping the research question [27], design of the project [28], how the research is conducted [12], and the resulting dissemination of study findings [19]. The most commonly reported impacts on service users include the acquisition of new skills and knowledge, increases in self-confidence, and feeling satisfied that they have made a difference [28].

However, less is known about the impact of coproduction on researchers themselves, or what researchers learn from working alongside community partners and service users [13]. Furthermore, little is known about the lessons learnt from coproducing specific types of research projects, such as feasibility/pilot studies incorporating an RCT design.

This paper aims to address identified gaps in the coproduction literature by providing a more rounded picture of co-production based on the experiences and learning outcomes of all those involved, such as community partners (i.e. staff members from the community mental health organisation), service users, and members of the research team involved in a feasibility/pilot study developing and evaluating an E-health Service for adults with depression, using an RCT design.

The following questions were developed:

1. What are the benefits of coproduction from the perspective of community partners, service users and health researchers?
2. What are the challenges of coproduction from the perspective of community partners, service users and health researchers?
3. What are the learning outcomes for community partners, service users and health researchers?

## Methods

### Research coproduction in context – The DES project

The research idea for this current study was formulated by the community partners at AWARE NI, a leading depression charity within Northern Ireland (NI) who approached research staff at Queen’s University Belfast (QUB) about working in partnership to develop and evaluate an online peer led support service for adults with depression.

One of the services offered by AWARE NI is a peer-led face-to-face support group for adults with depression. These groups take place on a weekly basis and are situated in both urban and rural areas in NI. AWARE NI were eager to expand this service to an online platform in order to increase their reach. From a provider perspective, they believed some people may be hesitant (fear of social stigma) or unable to attend face-to-face support for practical reasons (locality, lack of transport etc.).

The project had three distinct phases. Phase one focused on intervention development, and phases two and three focused on feasibility testing. Participants included adults (18+) seeking support for depression. The aim/objectives were to test the acceptability and feasibility of delivering a peer led support group intervention for depression using video conferencing technology. The findings from the feasibility study are currently being prepared and will be reported elsewhere.

### Sampling and recruitment

Using a purposive sampling approach, we interviewed researchers (*n* = 4), community partners (*n* = 3) and service users (*n* = 4) involved in this coproduction study. All 11 partners were contacted via email with an invitation to participate in interviews. The purpose of the interviews, an interview topic guide and consent form were attached to all email invitations. All invited partners were involved in the project from the beginning, including the early planning and design stages. Members of the research team frequently visited on AWARE NI’s premises (weekly basis) to discuss the project and developed working relationships with all partners over a 24-month period. Phase One included formal team meetings (*n* = 5) and workshops (*n* = 3) to develop the online service and study materials. Phase Two included internal testing of the service (*n* = 3) and additional workshops to develop the facilitator manual (*n* = 2). Phase Three included a number of observations associated with the intervention delivery (*n* = 4). Chairing responsibilities for team meetings were shared equally. Importantly however, the majority of contact between coproduction partners was informal e.g. phone or face-to-face conversations outside of formal meetings. This dynamic process provided valuable additional insight and ensured the project was constantly developing.

### Data collection

T.M. conducted semi-structured face-to-face (*n* = 4) and telephone interviews (*n* = 7) lasting between 30 and 45 mins with community partners, service users and members of the research team involved in the feasibility study. The interview topic guide focused on exploring key stakeholders’ experiences of coproduction; the impact coproduction had on the research itself and on them personally; what they felt were the key challenges; and conversely, what they felt were the key components for successful coproduction; along with any learning from this experience. The interview topic guide also explored how community partners felt about being involved in the research process, and how academics felt about their involvement (interview topic guide available upon request).

### Data analysis

After written informed consent was obtained from all participants, interviews were audio recorded, transcribed verbatim, and checked for accuracy by T.M. All interviews were conducted between November and December 2017. Thematic content analysis following Newell and Bernard’s framework [29] was used to analyse interview data. Key points emphasised by participants were coded under similar categories. These categories helped identify patterns in the data which led to key themes and sub-themes. A member of the research team, not involved in interviews, authenticated these themes by examining a random selection of interview transcripts. Any discrepancies in interpretations where discussed until consensus was reached. Finally, data analysis was confirmed by a community partner and service user. The GRIPP2 checklist [10] was used to ensure a comprehensive and transparent report of this research (Additional file 1).

### Reflexivity

As academics fundamentally supportive of community partners’ and service user involvement, we strove to maintain objectivity when conducting interviews and analysis. We were aware of the potential for participants to display social desirability bias [30], and stressed the importance for both positive and negative accounts of participants experiences prior to, and during interviews. We also searched for data that contradicted key emerging themes. Members of the research team interviewed for this aspect of the project were involved in the design phase. TM conducted the initial data analysis. TM was not involved in early development or design of the project and thus maintained a degree of objectivity. Co-authors were not involved in data analysis.

## Results

### What are the benefits of coproduction from the perspective of community partners, service users and health researchers?

#### Beyond transdisciplinary working – Developing community stakeholder networks

A key benefit of coproduction was the wider, longer term, impact of marrying the academic world with real-world community level service providers and users. The research team recognised the value of building on community partner relationships to identify and address real-world problems that often sit outside the health care system or health care practitioners’ peripheral vision. For example, linking with community partners provided researchers unique access to local community networks and thus knowledge of local issues that are often missed by statutory services. As a result, community partners and researchers were able to work together to identify other important research areas beyond the immediate research being conducted. By identifying other community stakeholder networks who addressed the needs of those in the community with chronic physical ill health, all partners identified the value of working together to address the unmet needs of those in the community who may develop depression due to physical ill health. This highlighted the importance of not only developing good working relationships with the research community, but also other community stakeholder networks. This joined-up approach to problem-solving across societal problems was seen as fundamental to moving research forward, relative to broader societal problems that exist outside of the clinical setting. This was viewed as going beyond the traditional transdisciplinary approach encouraged within research, by encouraging those from various community stakeholder groups to work together in developing further research ideas.

*In putting this [grant] application in and working on this [feasibility study], we’ve subsequently put another grant in with a couple of members, (research team member and community partner), to look at another area in terms of patients with stroke and mental health, an intervention for them, and one of the significant things in that has been to understand the assets that are in the community that can be used within a research setting (Researcher 3)*

#### Community partners’ involvement in data analysis and opportunities for co-authored papers

The research team viewed data analysis, the opportunity for co-authored papers and dissemination of findings as a vital part of coproduction work that is often overlooked. Vital in terms of producing and disseminating findings that are equally useful to community partners rather than for the academic community in isolation. Similarly, community partners felt that their lived experience of the condition the service is aimed to improve, makes them uniquely qualified to provide real-world interpretations, along with co-producing dissemination of findings in a user friendly format.

*The problem is, at the moment, there isn’t a method there that you can lift off the shelf to do it (involve community partners in analysing data), even though, at Queen’s, we are working on developing one. I think they [partners] need to be involved. I would like to see them (community partners) involved in the publication process as well (Researcher 4)*

*Having suffered from depression myself, I think we should have some people involved because they’ll have a better understanding when we get the feedback, maybe understanding it better, of exactly what…what people mean when they’re saying different things about it (online support group) to you, and we could interpret that better than someone who hasn’t got the experience of depression themselves (Service User 4)*

### What are the challenges of coproduction from the perspective of community partners, service users and health researchers?

#### Clear communication and resource planning – Marrying inputs with outputs

Interview data suggested that merging the two different worlds inhabited by each party also presented communication challenges. For example, researchers recognised that academic language can create a barrier to mutual understanding of research aims and expectations. Researchers, by virtue of their work, may be focused on longer term outputs in terms of further funding opportunities without taking into consideration the implications that may have on the ground for community partners in relation to resources and the capacity to implement long term goals.

*So, the kind of language around evidence and methodology, I think, takes a while for people to understand the various views and expectations on what that might look like. We talked about what the outcomes would be, but not about what the inputs would be to achieve those outputs in future. So, in other words, what would we need if we were to do another study, definitive study on this, to scale this up, what would AWARE need if they were to implement this in more consistent practice? (Researcher 3)*

*I would definitely get involved again because I think it (coproduction of research) has driven us to do this and to find out what we’ve found out so far. I think I would have to be more involved in the talk about the resourcing of it… I think I would also ask that a lot of the administration side of things would be taken by Queen’s, as you had to do, because I found… it was just not feasible for me to take that on. And nobody foresaw that that was the way it was going to happen, but I think that would have to be agreed before I would take it on again. (Staff 2)*

#### Time as a resource

Further support for the importance of resource planning was found in relation to the issue of ‘time’. Coproduction could at times slow down the research process when non-academic partners had other more pressing commitments. The research team also felt that the time pressures of meeting project deadlines presented challenges to meaningful coproduction throughout the entire research process, for example, in terms of time for training non-academic partners in data analysis techniques. Equally, time constraints were also a key challenge for community partners in terms of time to devote to the project on top of their normal workload. Community partners were working in a small organisation, which meant they did not have extra resources in terms of staff who could dedicate their time to the project. Therefore, the research team had to balance their desire for community partners’ full involvement with the practicalities of working with a small organisation with limited resources, so as not to overburden them.

*I think one of your main barriers is time because, obviously, you would like to do more with coproduction… ideally at each stage of the research project, you want to make sure that it’s in there. But, once an organisation starts to get involved in coproduction, other people can be asked to do a lot of things. So, in AWARE, they were also coordinating recruitment, they were delivering the intervention, there was maybe that sense at times that you could be putting a burden on them (Researcher 1)*

*I suppose we’re coming from two very different sort of backgrounds, and we look at probably different demands. I would think that, when Queen’s would be doing a piece of research, that that is your set piece of work. In terms of ourselves, we have other work as well, because, I have other competing demands in terms of this as well (Staff member 1)*

#### When two worlds collide – Understanding real-world implementation

Further challenges related to different ways of working for both parties. Interview data highlighted the tendency for research to be very rigid in relation to how things should be done to meet ethical guidelines and increase methodological rigour. However, this presented challenges in relation to the flexibility required for delivering a pragmatic support service for adults with depression. For example, face-to-face support groups, the model on which the online support groups were developed, involved a more flexible drop-in system of working where depressed individuals could attend as many groups as they wanted or needed to, for as long as they wanted or needed to. In the context of a feasibility/pilot study using an RCT design participants could only join one group per week for an eight-week period. Additionally, the RCT design meant that the control group had to wait 6 months before they could access the online service, and could not avail of face-to-face support from AWARE NI during this period. This raised concerns for community partners in relation to depriving people with depression from accessing the help they needed.

*I have to say that there were times when I felt that, a study and something as flexible as a support group working together…there were incompatibilities to that, the incompatibilities being that people don’t come to the support group for an eight-week period, they come for as long as they need it. They drop in and out of it as they need it. I did have some concerns of trying to kind of marry that, what was very much…a clinical trial really, with something that had to be very responsive (Staff member 2)*

*It’s probably just that yous were doing like the study, whereas we’re involved with the people themselves, with the problems. That was the one thing which I was concerned about at the start, was that a certain number of people will get the help and then a certain number of people aren’t getting the help, so there was a worry for me there that…what if these other people need, really need the help (Service User 4)*

### What are the learning outcomes for community partners, service users and health researchers?

#### Flexibility, pragmatism and methodological rigour

The research team had to rethink the design and ultimately had to abandon the RCT approach to introduce an element of flexibility for the project to work, which also helped community partners to see that the research team were prepared to try different approaches, and be responsive to real world implementation demands.

*Queen’s were incredibly flexible, and I really appreciated that… and as it became more and more evident that this wasn’t going to work in the way that it had been intended, that we had hoped it would work, or that Queen’s had hoped it would work, that their flexibility was great, and so now, what we’ve ended up with is we have two groups, at two different times, that people can come into, and they can swap if they can’t make one group or the other, and that is how our support groups work (Staff member 2)*

This approach proved popular with the service provider and reinforced the concept of equal ownership among all parties. However, there was a sense of disappointment among the researchers as they considered the impact this change would have on the quality of output (potential journal publication). This is further evidence of competing priorities within coproduced research as journal publications are often a proxy for impact with research excellence frameworks. As such, members of the research team felt they were judged more on methodological rigour whereas service providers focused on the outcome of the intervention. While the former is important in order to reduce potential bias in the reporting of results, a coproduced approach to RCT development did appear to ‘muddy the waters’. Particularly as RCTs have a clear set of guidelines and procedures in which to adhere.

#### Getting it right from the start: Seeing each other as assets

RCT’s are a complex research design and potentially ‘off-putting’ for those less au fait with the process. Having community partners ‘involved’ in the entire research process appeared to be a way of mitigating against this. This was established from the outset through the joint development and preparation of a successful funding application through a scientific funding stream. This commitment to practising the ethos of coproduction from the embryonic stage of the research process created a sense of ownership of the project for community partners.

*I think what’s important here is that Queen’s and AWARE both feel that there’s an ownership, there’s a sense of ownership, and it is collaborative. But it’s not to say that, you know, this is Queen’s project or this is AWARE’s project – it belongs to both of us (Service User 1)*

*The challenge is trying to think of ways of increasing their [community partners] ownership and increasing their control. I think it starts from the initial [research funding] bid (Researcher 4)*

However, despite researchers’ good intentions in relation to having partners involved in all aspects of the research process, it was apparent from partners reports that there was a need to balance this out with what level of involvement partners really want, as too much involvement could be experienced as overwhelming.

*I know we have to know how the process works and have a feeling of all the parts of it, but there was some bits of it, I think, where just…it was sort of heavy and overload, at times (Staff member 1)*

As such, it is important to recognise that, for some, the various complexities associated with RCT development and implementation do not need to be shared. Nonetheless, the information should be made easily available to those who want it (randomisation, data analysis, fidelity etc.)

#### Identification of team strengths and expertise

A sense of ownership appeared to be fostered by a sense of mutual respect for each other’s strengths. From the research team perspective, community partners’ unique knowledge in terms of service delivery and the nature of depression added quality and credibility to the research that theory, and academic knowledge alone could not provide. From a community partner and service user perspective, researcher involvement provided structure, support and a sense of weight to the project.

*As a researcher, you have your background knowledge, you have your own theory, you look through papers, you look through best practice, but it’s about having that additional perspective from people actually who are delivering a service or who are using the service (Researcher 1)*

*I think that it added a lot of, really structure and organisation. The partnership with Queen’s, it really helped to get things up and running and it was kind of like Queen’s were like the brains of it and AWARE were kind of like the heart (Service User 3)*

#### Being present

As outlined in the previous section, it was evident that both the research team and community partners shared key decision making and responsibility for the project at all key stages of the research process. This sense of ‘we’re in this together’ appeared to be enhanced by close contact between the research team and community partners throughout the project. This close contact extended beyond research team meetings, to include having a visible research team presence within the community partner’s organisation.

*We were in and out of the office a lot, so when they were setting up things, we went over and had a try, had a bit of a laugh and stuff like that. It’s about …feeling more like you’re actually part of a team. I think if you’re only having a meeting every three months or something, that’s not going to work so well, but if you’re over there regularly… So, I think, you know, with coproduction, I think it’s really about taking every opportunity you can actually to meet up with that team and going along maybe to some of their events and being present really, you know, being available when they need you (Researcher 1)*

**Fig. 1:**
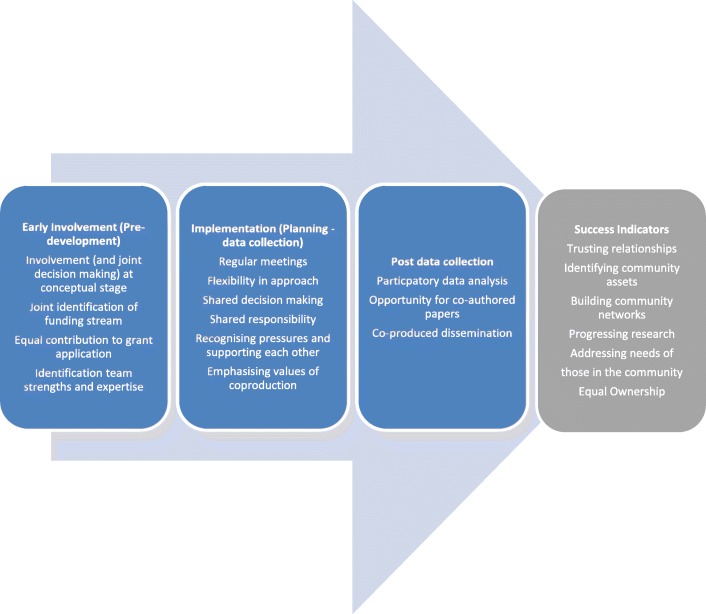
Three phase implementation model for coproducing research with community partners using a feasibility/pilot RCT design

Figure 1 draws these findings together to provide a visual picture of the lessons learned and potential process of implementation for coproducing research with community partners using a feasibility/pilot RCT design. The figure was developed in collaboration with coproduction partners.

## Discussion

A number of research projects have recently emerged reporting on coproduction of research with healthcare clinicians, managers, patients and members of the public [31], patients and carers [25], and with community partners [4]. This paper attempts to add to this emerging knowledge base in relation to exploring the experiences of *all* those involved in a coproduced research project using a feasibility/pilot RCT design.

### Benefits of coproduction

A number of benefits in relation to co-production were identified throughout the process, this included additional ‘insider’ knowledge that strengthened the team’s ability to address real-world problems. However, getting to this point is not simply a matter of inviting potential end users to become ‘involved’ in research. In order to maximise the benefits from coproduction partners, one must consider a number of important factors, namely (1) early involvement at the pre-development stage, including contributing to the scientific grant application; (2) early identification of team strengths and expertise from the outset; (3) regular team meetings and contact (formal or informal) among coproduction partners; (4) a flexible and pragmatic approach to research design (particular within RCTs); (5) shared decision making and responsibility and (6) recognition of ‘other’ pressures and providing support to each other. Findings also suggested further scope for involving community partners in data analysis and dissemination through co-authored papers. In combination, these factors facilitated a number of important processes that enabled trusting relationships to develop. The benefits of this will continue long after the current project has ended.

### Challenges and boundaries

Findings also suggested a number of challenges when coproducing research. While emerging evidence tends to highlight the need for and benefits of involving practitioners, patients, carers and/or community partners in research, there is also growing debate on the dangers of taking coproduction too far [32, 33], and we have recognised some of these limitations when discussing the challenges of this approach. Those who have provided an opinion appear to argue in favour of maintaining boundaries [34–36]. Orr et al. [35] argue for a model of coproduction ‘based on the idea of mutual recognition in which both sides retain their own integrity’ [p. 202]. One area that continues to be an issue is in relation to partners’ involvement in data analysis - a vital, but often overlooked part of the coproduction process. We believe the onus is on academics to find innovative ways of providing adequate training in data analysis techniques that take due account of community partner’s work/life time constraints.

Interestingly, some literature suggests that trust increases as boundaries blur. [37]. As such, it is perhaps more about being aware of which boundaries are safe to blur, and which boundaries are best maintained. For example, if we take the lessons from previous research [4] and our current findings, blurring the boundaries in relation to equal power sharing and co-governance helps build trust and in turn appears to facilitate sustainable relationships; whereas blurring the boundaries between each communities’ areas of expertise may be more damaging. We believe a key learning point is not to approach the ethos of coproduction as a tick box exercise in terms of having non-academic partners do X, Y, Z, but rather focus on having negotiations from the outset in relation to who is comfortable taking on which roles in joint recognition of the diverse range of skill sets and knowledge each person in the partnership brings to the table. As further stressed by Boyle et al. [16], both research and experience clearly demonstrate that there is no precise guidance, toolkit or manual for coproduction.

### Implications for coproduced RCT designs

In relation to implementing a coproduced RCT design the data revealed a number of important considerations.

Firstly, the difficulties of developing and delivering a service within the constraints of an RCT design were highlighted. Although these concerns appear specific to this project, they may be transferable to others embarking on the same research design. For example, as found in previous research on “natural” self-helping practices, real-world implementation can actually be hindered in the context of a controlled research setting [38]. This dilemma has been discussed in previous literature on implementation science in relation to how to deliver an intervention project that is relevant in real-world settings without sacrificing scientific rigour [39]. As these authors have pointed out, “the more controlled the setting is, the more artificial and less directly informative about impact in real-world settings the participant behaviors are” [p 2]. This is never more apparent than when working in partnership with those in the community [39], whose biggest concern was in relation to those in the control group having to wait 6 months before accessing support. This raised a number of ethical concerns for partners and revealed the importance of building knowledge of research design during the process, albeit at an accessible level and one that does not create additional boundaries.

Secondly, some argue that RCTs are too slow, costly, and unable to adequately capture the effectiveness of real-world complex interventions [40], and as such not suitable for extending knowledge on health care delivery [41], others argue that these challenges can be overcome by using a pragmatic trial approach [39]. For example, a pragmatic trial design aims to determine effectiveness of an intervention under usual conditions so the intervention remains the same as it would if delivered alone with no evaluation [42]. Pragmatic trials also allow for more flexibility which we found was extremely important when working with community partners. However, even a pragmatic trial approach does not completely address all the concerns of community partners highlighted in the findings of this study. For example, although pragmatic trials allow for more flexibility in terms of co-interventions (interventions delivered alongside the intervention being evaluated, which are usually restricted so that any change in participant outcomes can only be attributed to the intervention being evaluated), restrictions still apply in relation to co-interventions if they are likely to dilute the intervention effect. There are however a number of approaches which could help overcome this challenge and reassure community partners that no one suffering from the condition an intervention aims to alleviate will be deprived of support. For example, superiority trials aim to determine a clinically significant difference between two interventions; equivalence trials aim to determine if a new intervention is neither better nor worse than an existing intervention; and lastly, a non-inferiority trial aims to determine if a new intervention is, as the name suggests, not inferior to an existing intervention [43]. Whichever approach is used, everyone, including those in the intervention and co-intervention group, receive support of some sort.

Thirdly, there were issues with standardising the intervention in such a way as to provide meaningful data on likely outcomes. This issue appeared to clash with some of the core values of the service currently delivered by AWARE NI i.e. that individuals must attend for a certain number of weeks (8-weeks) and then the service is withdrawn/stopped. A solution to this issue was found through offering follow up face-to-face support for those finishing the online groups. However, this had implications for the three and six-month evaluation periods. As such, for this study the RCT design (potentially) created a tension between data quality and service user well-being. However, the relationships developed in the early stages of this project were vital to ensuring workable solutions to this issue, with both the research team and project partners comfortable to discuss the advantages and disadvantages of approaches.

An important issue here was the use of academic language e.g. control, potential confounders etc. For example, members of the research team were acutely aware that academic language can cause confusion around research aims and expectations, particularly if the designs are relatively complex. This reaffirms the importance of using layman’s terms from the outset, incorporating resource planning at the funding application stage. Inputs required from community partners to produce the longer term research outputs need to be clearly defined; all-the-while taking into account community partners concerns around resources and capacity, and how they can be best supported by academic partners.

Study findings are summarised and developed further within the implementation model listed above (Fig. 1). This may prove useful to others when coproducing research with community partners in general, along with key factors to consider when coproducing a feasibility/pilot randomised controlled trial (RCT). The model expands on the work of Boyle et al. [16]. The first phase of the model incorporates two of Boyle et al. principles of coproduction which we have adapted slightly in light of our findings. For example, Boyle et al. recommend recognising people (with people meaning the public) as assets, which we feel still implies an implicit power imbalance as this involves a one way change in mind-set; namely that of public service providers. We have adapted this to include recognition of *each other* (both academic and non-academic partners) as assets to redress this power imbalance and highlight the importance of *mutual* recognition of *each other’s* unique abilities which we feel is more reflective of equal power-sharing among all parties involved**.** The model draws together learning from our findings in relation to the key steps and potential bumps in the road when coproducing research with community partners using a feasibility/pilot RCT design. Success indicators outline how consideration of these important factors have the potential to lead to longer term outcomes in relation to progressing research through identifying and addressing community level needs.

## Strengths and limitations

All interviewees were involved in this coproduced research which may have influenced their views. Furthermore, this study’s findings are based on a small sample of researchers, community partners and service users in relation to only one project, which limits their generalisability to other settings and conditions. However, we believe, having reviewed the relevant literature, that providing a more rounded picture of community partners, service users and researchers experiences of coproduction provides new insight regarding RCT coproduction that extends learning from Boyle et al.s’ [16] coproduction framework.

To our knowledge this is the first paper to address coproduction within a feasibility/pilot RCT design which provides some transferable lessons to others embarking on similar projects.

## Conclusion

Our three-phase coproduction model has extended learning from previous research in relation to the key steps for consideration when coproducing research with community partners and service users in general, along with key factors to consider when coproducing a feasibility/pilot RCT. It is important that each party recognise *each other* as assets so everyone involved sees the value in each other’s contribution to the research. All partners should be involved in grant writing, data analysis and dissemination to increase joint ownership and neutralise power imbalances. Resource planning is vital to ensure that outputs are realistic and achievable in light of the inputs required by community partners, and clear communication is required to ensure everyone understands the roles and expectations of all involved. Finally, when conducting a feasibility/pilot using an RCT design, special consideration should be given to real-world implementation. As noted previously, this model should be used for considering the key factors of coproduction rather than as a prescriptive formula, as different contexts may require different approaches. We urge others to test this proposed model more widely in different areas of coproduced research.

## Additional file

Additional file 1: GRIPP2 long form. (DOCX 14 kb)

## Abbreviations

GRIPP: Guidance for Reporting Involvement of Patients and Public
NI: Northern Ireland
PPI: Patient and Public Involvement
QUB: Queen’s University Belfast
RCT: Randomised Controlled Trial
UK: United Kingdom
