# Supplementary material for: Coproduction for feasibility and pilot randomised controlled trials: learning outcomes for community partners, service users and the research team
Source: Res Involv Engagem. 2018 Oct 8;4:32. doi: 10.1186/s40900-018-0116-0 (PMC6174568; doi:10.1186/s40900-018-0116-0)
Supplement: Supplementary file 1 — GRIPP2 long form. (DOCX 14 kb) [file 40900_2018_116_MOESM1_ESM.docx]

**Table 1 GRIPP2 long form**

| Section and topic | Item | Reported |
| --- | --- | --- |
|  |  | on page |
|  |  | No |
|  |  |  |
| Section 1: Abstract of paper |  |  |
| 1a: Aim | Report the aim of the study | 2 |
| 1b: Methods | Describe the methods used by which patients and the | 3 |
|  | public were involved |  |
| 1c: Results | Report the impacts and outcomes of PPI in the study | 3 |
| 1d:Conclusions | Summarise the main conclusions of the study | 3-4 |
| 1e: Keywords | Include PPI, “patient and public involvement,” or alternative | 4 |
|  | terms as keywords |  |
| Section 2: Background to paper | |  |
| 2a: Definition | Report the definition of PPI used in the study and how it | 4 |
|  | links to comparable studies |  |
| 2b: Theoretical | Report the theoretical rationale and any theoretical | 5-6 |
| underpinnings | influences relating to PPI in the study |  |
| 2c: Concepts and | Report any conceptual or theoretical models, or influences, |  |
| theory development | used in the study |  |
| Section 3: Aims of paper |  |  |
| 3: Aim | Report the aim of the study | 7 |
| Section 4: Methods of paper |  |  |
| 4a: Design | Provide a clear description of methods by which patients | 7-8 |
|  | and the public were involved |  |
| 4b: People involved | Provide a description of patients, carers, and the public | 7-8 |
|  | involved with the PPI activity in the study |  |
| 4c: Stages of involvement | Report on how PPI is used at different stages of the study | 7-8 |
| 4d: Level or nature of | Report the level or nature of PPI used at various stages | 7-8 |
| involvement | of the study |  |
| Section 5: Capture or measurement of PPI impact | |  |
| 5a: Qualitative evidence | If applicable, report the methods used to qualitatively |  |
| of impact | explore the impact of PPI in the study |  |
| 5b: Quantitative evidence | If applicable, report the methods used to quantitatively |  |
| of impact | measure or assess the impact of PPI |  |
| 5c: Robustness of | If applicable, report the rigour of the method used to |  |
| measure | capture or measure the impact of PPI |  |
| Section 6: Economic assessment | |  |
| 6: Economic assessment | If applicable, report the method used for an economic |  |
|  | assessment of PPI |  |
| Section 7: Study results |  |  |
| 7a: Outcomes of PPI | Report the results of PPI in the study, including both | 10-19 |
|  | positive and negative outcomes |  |
| 7b: Impacts of PPI | Report the positive and negative impacts that PPI has | 18-19 |
|  | had on the research, the individuals involved (including |  |
|  | patients and researchers), and wider impacts |  |
| 7c: Context of PPI | Report the influence of any contextual factors that | 10-19 |
|  | enabled or hindered the process or impact of PPI |  |
| 7d: Process of PPI | Report the influence of any process factors, that | 10-19 |
|  | enabled or hindered the impact of PPI |  |
| 7ei: Theory development | Report any conceptual or theoretical development in | 19-20 |
|  | PPI that have emerged |  |
| 7eii: Theory development | Report evaluation of theoretical models, if any | 21-14 |
